# Management of Infections in Palliative Care Patients at the End-of-Life and Active Process of Death: A Brazilian Retrospective Study

**DOI:** 10.1089/pmr.2024.0005

**Published:** 2024-08-02

**Authors:** Isabela Fernandes de Aguiar Tonetto, Angelita Maria Stabile, Dieyeni Yuki Kobayasi, Rita de Cássia Quaglio, Ana Carolina de Souza, Fabiana Bolela

**Affiliations:** Enfermagem Fundamental, Escola de Enfermagem de Ribeirão Preto—Universidade de São Paulo, Ribeirão Preto, Brazil.

**Keywords:** dying process, end-of-life care, infection, nursing, nursing care, palliative care

## Abstract

**Background:**

There is a lack of specific studies on the management of infections in patients receiving palliative care (PC) in the final stages of life and during the active process of death, related to specific nursing care. There is clinical and social importance as patients in PC represent a vulnerable population, and adequate management of infections is crucial to improve quality of life and the experience of comfort.

**Objective:**

This study analyzed how infections are managed in patients undergoing PC at the end-of-life and in the active process of death in two hospital health services.

**Design:**

This is an observational, analytical, and retrospective study.

**Settings:**

Data collection took place in two hospitals that assist individuals who are hospitalized under PC, located in Brazil, in a city in the interior of the state of São Paulo.

**Measurements and Results:**

The sample consisted of 113 medical records, in which the oncological diagnosis was the most prevalent. There was a predominance of infection diagnoses based on the patient’s clinical symptoms, the main focus being the pulmonary, in individuals at the end-of-life. The management of infection in the study sample occurred through care and procedures that generate physical discomfort, however aiming at relieving symptoms. Such findings must be documented, as they invite us to reflect on our practical attitudes and what it means to be comfortable for these people, making it possible to incorporate this information into the design of interventions focused on enhancing the experience of comfort.

## Introduction

The World Health Organization defines palliative care (PC) as an approach that promotes quality of life for patients (adults and children) and their families who face life-threatening illnesses, through prevention and the alleviation of suffering whether of a physical, psychological, social, or spiritual nature.^1^

End-of-life represents the last six months of a person with a serious, life-threatening illness.^2–4^, In this phase, the body enters a state of intense catabolism, owing to the action of cytokines, which promote fat loss, followed by muscle loss. This process generates a situation of cachexia, in which the loss of vitality will lead to the gradual failure of multiple organs.^5^

The period referring to the last 72 hours or days before death is called the active process of death.^6^ In this phase, the individual’s physiological functions progressively reduce,^7^ and there is a worsening of signs and symptoms.^8,9^

People undergoing PC are hospitalized owing to clinical decompensations, and hospitals need to develop and/or improve evaluations and care planning protocols for these stages of life.^10,11^

Patients undergoing PC often have significant underlying medical conditions that can compromise their immune system and ability to fight infections. It is common for these people to present infection owing to failure of the anatomical barrier triggered by chemotherapy, radiotherapy, dehydration, xerosis, decrease in the function of neutrophils, and cellular immunity, in addition to cachexia.^12^

Clinical evidence on the management of infections and the indication or not of antibiotics in the end-of-life phase and active death process are scarce, and the results are divergent.^13–17^ The use of antimicrobials does not prevent the progression of cancer or advanced dementia; on the contrary, it may increase survival time and not contribute to the control of symptoms related to the infectious process.^17–19^

Symptoms of an infectious disease can vary widely, and the approach to treating an infectious disease in patients undergoing PC is often focused on symptom relief and patient comfort. This may include using antibiotics to treat bacterial infections, antifungals for fungal infections, and antivirals for viral infections, if appropriate. However, in some cases, treatment may be limited to avoid burdening the patient with invasive or aggressive interventions.^13,14^

In addition to antibiotic therapy, some care provided, such as nursing procedures related to nutrition, in patients at the end-of-life, are not beneficial, as the swallowing reflex, especially of liquids, is reduced.^6^ The same observation applies to the use of therapies with curative purposes for tegumentary lesions.^20–23^

The nurse, as part of the multidisciplinary team, through systematized assistance, must evaluate and prescribe specific individualized care for the control of the signs and symptoms observed as altered, whether pain, nausea, dyspnea, or fatigue, among others.^24,25^

An intervention is defined as any treatment based on judgment and clinical knowledge that a nurse performs to improve outcomes for the individual under their care.^26^ Despite the importance and recurrent relevance of this theme in hospital environments, there are a few studies regarding the management of infections in patients undergoing PC regarding nursing interventions. This study analyzed how infections are managed in patients undergoing end-of-life PC with an active death process in two hospital services.

## Methods

### Location and casuistry

The research was carried out by analyzing the medical records of patients undergoing PC treatment at two public hospitals located in the interior of the state of São Paulo, Brazil. These hospitals are part of the Unified Health System.

One of the hospitals is a secondary level of care, intended for scheduled, nonemergency hospitalizations. The other hospital service has complexity of care at the tertiary level, and this place is intended for care in situations of illness with a high risk of death. Both hospitals have teams that assist, in a specialized PC way, patients in hospital wards, caring for each individual according to their pathology and symptoms. In the secondary hospital, patient assessment is continuous horizontally; however, in the tertiary hospital, the PC team carries out specific assessments on the day, and the daily care of individuals is carried out by nonspecialist professionals. Even though they are in different physical locations, these services interact through the discussion of clinical cases and are part of the municipality’s PC support network.

### Study participants

The study included medical records of patients undergoing PC at the end-of-life or an active death process, of both sexes and aged over 18 years and with a diagnosis of infection, regardless of the focus or microorganism, from January 1, 2019 to December 31, 2019. Only the last infection event recorded in the patient’s medical record before death was analyzed.

The focus event of the study was the occurrence of the infection and how it was managed, from its diagnosis to the clinical outcome (death) in the participant’s last hospitalization in the defined collection period. An individual is considered to be at the end-of-life when the life prognosis is less than six months, associated with the diagnosis of a serious and life-threatening illness.^4^ When, in an active process of death, this individual experiences the last 72 hours of life, associated with a reduced level of consciousness and reduced urinary output.^6^

Nursing records made in the study participants’ medical records were considered to evaluate the reduction in the level of consciousness and reduction in the number of diaper changes (zero or one change per day), with urinary output, in association with the participant’s length of stay of study.

### Study variables

The information of interest to the study was collected directly from the medical records of patients undergoing PC who presented a diagnosis of infection. In addition to sociodemographic and general clinical characteristics, information regarding the management of the infection was collected until the clinical outcome of the infection or death.

The data collection form contained five parts: I—Identification, II—Diagnosis, III—Diagnosis of Infection, IV—Outcome of the case, and V—Records of the Nursing Team.

Nursing interventions were categorized according to the Nursing Interventions Classification for data description and analysis.^26^ Identification of the infection was based on the record of suspected or confirmed infection by the medical team in the patient’s medical record.

### Procedure for analyzing and processing data

Data were initially entered into a simple Microsoft Excel table and then transferred to the IBM Statistical Package Social Science (SPSS) version 23.0 for Windows. The data obtained were analyzed descriptively in terms of frequency (nominal variables), central tendency, and dispersion (numerical variables).

### Ethical aspects

This project was approved by the Research Ethics Committees of the University of Ribeirão Preto College of Nursing from São Paulo under opinion No. 30908220.90000.5393 and from the Hospital das Clínicas of the Faculty of Medicine of Ribeirão Preto, University of São Paulo, under opinion No. 30908220.9.3001.5440.

The waiver of the Free and Informed Consent Form was requested, as the collection was based on data from medical records and the patients had died at the time of collection.

As this is research involving data from human beings, the confidentiality of the patient’s identity was guaranteed to preserve their dignity and vulnerability, according to Resolution n. 466/2012 of the National Health Council from Brazil.

## Results

The study population consisted of 113 medical records, with 58 (51.3%) being male and 55 (48.7%) female, of which 71 (62.8%) belong to a secondary institution and 42 (37.2%) to a tertiary institution. There is a prevalence of 95 (84.0%) individuals over 60 years old, whereas 18 individuals (16.0%) were under 60 years old.

In the total sample of 113 individuals, only 2 did not have any comorbidity. Therefore, to calculate the frequency of comorbidities in the sample, the total number of 111 individuals was used, of which the most frequently identified comorbidity was arterial hypertension (73; 65.7%), followed by acute renal failure (28; 25.2%), heart failure (26; 23.4%), diabetes mellitus (15; 13.5%), and liver failure (4; 3.6%).

The main prevalent severe chronic disease diagnosis was oncology (63 individuals; 55.8%), followed by neurology (35 individuals; 30.9%), and organic insufficiency (15 individuals; 13.3%). At the time of diagnosis of the infection, 77 individuals (68.1%) were at the end-of-life and 36 (31.9%) were in the active death process. The time from the diagnosis of the main serious chronic disease to being linked to PC had a median of 10 months, ranging from 0.0 to 230.9 months, and the mean time in PC was six days, with a range from 0.0 to 20.3 months, from linking to the outcome of death.

Regarding the identification of the infection, there was a predominance of the clinical diagnosis with 69 cases (61.1%), followed by laboratory examination (59; 52.2%), and imaging exam (27; 23.8%), with the main focus being the lung with 73 (64.6%), followed by urinary (19; 16.9%), cutaneous (10; 8.9%), and others (11; 9.7%).

Antimicrobial therapy was prescribed for 110 (97.3%) of the individuals, predominantly indicated by the team responsible for the case (59; 53.6%), followed by the PC specialist, with 51 cases (46.4%). The main pharmacological classes of antimicrobials were cephalosporins (48; 59.5%) and penicillins (32; 53.8%); in all, 10 different classes of prescribed antibiotics were identified. The main route of administration was intravenous, against oral, with 7 cases (6; 4%).

In addition to antimicrobial therapy for managing the infection, other interventions were prescribed for patients, with emphasis on oxygen therapy (*N* = 96; 85%). However, hemotherapy (*N* = 3; 2.7%) and interventional therapies, such as paracentesis and thoracentesis (*N* = 2; 1.8%), were less recommended.

It is observed that the median age and follow-up time by the PC team were not different between individuals at the end-of-life and active death process; however, the duration of antibiotic therapy and the time elapsed from the beginning of death to death were statistically lower in individuals in an active death process.

The median time elapsed from the diagnosis of infection to the outcome of death was nine (minimum = 4; maximum = 34) days for individuals in the end-of-life stage and two (minimum=zero; maximum = 3) days for individuals in an active dying process.

In the records of nursing interventions, a higher frequency of measuring vital signs of 88 individuals (85.9%) was observed, followed by skin care 85 individuals with a frequency of 87.9%, and changing positions, 83 cases with a frequency of 90.0%. It is noted that interventions related to pain control and family support were registered with a lower frequency, 49 cases (55.3%) and 10 cases (11.0%), respectively, than the previous variables.

It is observed that there is a predominance of diaper-changing procedures (*N* = 108; 145.8%) and venipuncture for the insertion of a peripheral catheter (*N* = 108; 98.3%) and for the collection of laboratory tests (*N* = 85; 93.3%). It is noteworthy that the same patient may have undergone a given procedure more than once during hospitalization.

Nasoenteric tube insertions were performed in 59 cases, constituting 52.5% of the individuals, and the nasogastric tube insertions were carried out in 7 cases, constituting 6.2% of the studied population. Airway aspirations were conducted in 58 cases, with a percentage exceeding 100% (151.0%) potentially indicating multiple occurrences per patient, and indwelling bladder catheterizations were performed in 42 cases, representing 37.2% of the individuals, and against relief bladder, catheterizations were conducted in 11 cases, reflecting a percentage of 9.8%.

Physical restraints were used in 1 case, representing 1.9% of the individuals in PC diagnosed with infection.

## Discussion

This study investigated infection management in end-of-life patients receiving PC at secondary or tertiary hospitals. Findings revealed a median of 10 months from the diagnosis of the main chronic disease to the initiation of PC, with a median follow-up of 6 days until death. These results suggest limited access to PC, even in major health centers, with most patients receiving care shortly before death.

Late referral to PC services is driven by misconceptions among team members, patients, and family, who mistakenly associate PC with treatment discontinuation and reserve it for those in the end-of-life stage.^27^ Furthermore, inadequate training of health care professionals and missed opportunities to address PC perpetuate the stigma surrounding it.^28,29^

To identify infections, clinical diagnosis was the primary method, often leading to empirical antimicrobial therapy, followed by laboratory and imaging examinations. Notably, 97.3% of the patients in this study were prescribed antibiotics. This aligns with literature suggesting that patients with cancer experiencing fever post-chemotherapy should undergo blood cultures and receive empirical antibiotics until afebrile for 48–72 hours.^30^ A study at the University of Michigan Health Service involving 131 patients with advanced cancer in outpatient care found that 70 patients received antimicrobials, with 54 receiving empirical therapy.^31^

A study from Australia involving 137 patients showed that 86 received antibiotics at the end-of-life and, of these, 44.2% continued using them during the active process of death. The most prescribed pharmacological class was beta-lactams (41.9%), followed by cephalosporins (15.4%). The most common site of infection was the lungs (32.8%). Of the 86 patients prescribed antibiotics, 83 (96.5%) received antimicrobials intravenously.^32^

In the present study, pulmonary infection emerged as the primary focus of infection, with intravenous administration being the most common method for antibiotic delivery. This choice is favored owing to the need for rapid therapeutic effects, given the duration of the infection.^33^ However, intravenous administration, while effective, carries inherent risks, with phlebitis and sepsis being the most prevalent concerns associated with this invasive procedure.^34,35^

The pharmacological class of antibiotics most commonly used in these patients was cephalosporins, followed by penicillins, with a median treatment duration of six days. Despite being under the care of PC teams toward the end-of-life, a significant proportion of patients, ranging from 63% to 90%, continued using antibiotics in the last week of life.^14,32^ This persistent antibiotic use, even in the face of impending death, underscores the ongoing debate regarding antibiotic indications at the end-of-life,^16,36^ with a lack of established guidelines for antimicrobial administration in PC settings.

A retrospective study conducted with 459 individuals in the terminal stages of illness at a general hospital in Singapore revealed that the continuation of antimicrobial therapy at the end-of-life resulted in a reduction in survival time by one day compared with those where antimicrobial therapy was suspended 48 hours before death.^37^

Studies on prolonging survival with antibiotics are divergent.^19^ Two studies carried out on the use of antibiotics in patients undergoing PC, with a primary diagnosis of advanced cancer, showed no difference in survival time.^38,39^ However, other studies have shown that antibiotics prolong survival from days to weeks.^13,37,40,41^

The study prompts reflection on antimicrobial therapy in patients in an active dying process, as it may not alter the expected survival time. In addition, oxygen therapy, used in 85% of cases, serves as the primary adjunct therapy for respiratory discomfort relief. However, its efficacy relies on the presence of hypoxemia. Nonpharmacological approaches, such as optimizing room airflow, can also alleviate breathlessness.^42^

The critical and individualized multidisciplinary assessment of patients in PC facing end-of-life infections and an active dying process poses a significant challenge. Although literature lacks consensus on antimicrobial indication and duration, it emphasizes their role in symptom management. Vital sign monitoring supersedes the collection of biographical and psychosocial data in patients undergoing PC,^43^ as it aids in prognosticating survival time.^44,45^

Regarding changing diapers, skin care, and changing positions, the literature highlights the importance of these procedures, at the time of bathing, for comfort, reduction of agitation, aggressiveness, and stress at the end-of-life,^46^ aiming to provide a “good death.”^47^

The relief of suffering, through adequate control of the symptoms related to the infection of an individual who lives with a serious and life-threatening illness, must involve specific nursing actions and interventions.^48^ Symptoms such as fatigue, pain, and discomfort are common, and the nurse’s clinical assessment should include everything from the adequacy of the environment in which the individual is to the choice of the best device and the route of administration for drug therapies, in order to reduce damage and ensure effectiveness in controlling current symptoms.

In addition to venipunctures for drug administration and sample collections, airway probes and aspiration were frequently performed procedures in this analysis. Several studies highlight the discomfort associated with procedures such as urinary catheterization,^49,50^ nasogastric/nasoenteral catheterization,^51,52^ and nasotracheal aspirations.^53^

Individuals receiving PC are exposed to wrong procedures and unwanted experiences during the care process by health teams. Many doctors choose to prescribe medications and procedures fearing that the noncontinuity of treatments will make them vulnerable to families and justice itself.^54^

No clear evidence was found in the literature about the benefits of invasive procedures to ensure adequate control of symptoms and relief of suffering in the evaluated population. What is known is that in hospital environments, individuals are rarely able to exercise their autonomy.^55,56^

Nurses must advocate for the individuals to whom they provide care and thus ensure, through health education, the clarification of the process and the objectives of care, aiming at patient autonomy and the orthothanasia.

As a limitation of the present study, we highlight the fact that it was carried out in only one Brazilian municipality, which may not reflect the reality of managing infections in other locations. It is also difficult to discuss the data, as there are few published studies aimed at managing infections in individuals undergoing PC.

## Conclusion

Given the above, infection management in palliative patients at the end-of-life and active death process relies on symptom relief through care and procedures. Notably, there’s extensive antibiotic prescription, even for those in active death and a high frequency of invasive procedures. Documenting these practices is crucial for reflection on our care approaches, understanding the comfort of the patient, and informing targeted interventions to enhance the comfort experience.

## References

[1] WHO. guideline on self-care interventions for health and well-being. World Health Organization: Geneva, Switzerland; 2021. 34370426

[2] Silva RR, Massi G de A. Trajetória dos Serviços de Cuidados Paliativos no Brasil: Aspectos históricos e atuais. RSD 2022;11(11):e222111133545.

[3] Kira CM. Assistência às últimas horas de vida. In: Manual da Residência de Cuidados Paliativos (Carvalho RT. ed.) Manole: Barueri; 2018.

[4] Tatum PE. End-of-life care: Hospice care. FP Essent 2020;498:26–31. 33166104

[5] Luo J, Chen JJ, Deguzman C, et al. Symptoms and palliative care needs of pancreatic adenocarcinoma patients. J Palliat Med 2014;17(6):640–641. 24875242 10.1089/jpm.2014.0056 PMC4094017

[6] Mori M, Morita T, Bruera E, Hui D. Prognostication of the last days of life: Review article. Cancer Res Treat 2022;54(3):631–643. 35381165 10.4143/crt.2021.1573 PMC9296934

[7] Hwang IC, Ahn HY, Park SM, et al. Clinical changes in terminally ill cancer patients and death within 48 h: When should we refer patients to a separate room? Support Care Cancer 2013;21(3):835–840; doi: 10.1007/s00520-012-1587-4 22956193

[8] Hospice patients alliance–signs of approaching death [Internet]. Hospicepatients.org; 2019. Available from: https://hospicepatients.org/hospic60.html

[9] Randich A. Oxford textbook of communication in oncology and palliative care. J Palliative Med 2020;23(1):150–150.

[10] Arcanjo SP, Saporetti LA, Curiati JAE, et al. Clinical and laboratory characteristics associated with referral of hospitalized elderly to palliative care. Einstein (São Paulo) 2018;16(1):eAO4092; doi: 10.1590/S1679-45082018AO4092 29694607 PMC5968794

[11] Rodríguez-Acelas AL, de Abreu Almeida M, Engelman B, Cañon-Montañez W. Risk factors for health care–associated infection in hospitalized adults: Systematic review and meta-analysis. Am J Infect Control 2017;45(12):e149–e156. 29031433 10.1016/j.ajic.2017.08.016

[12] Hamilton KW, Fishman NO. Antimicrobial stewardship interventions: Thinking inside and outside the box. Infect Dis Clin North Am 2014;28(2):301–313; doi: 10.1016/j.idc.2014.01.003 24857395

[13] Furuno JP, Noble BN, Fromme EK. Should we refrain from antibiotic use in hospice patients? Expert Rev Anti Infect Ther 2016;14(3):277–280; doi: 10.1586/14787210.2016.1128823 26641527

[14] Wi YM, Kwon KT, Hwang S, et al. Use of antibiotics within the last 14 days of life in Korean patients: A nationwide study. J Korean Med Sci 2023;38(9):e66; doi: 10.3346/jkms.2023.38.e66 36880107 PMC9988432

[15] Oh DY, Kim JH, Kim DW, et al. Antibiotic use during the last days of life in cancer patients. Eur J Cancer Care (Engl) 2006;15(1):74–79; doi: 10.1111/j.1365-2354.2005.00603.x 16441680

[16] Stiel S, Krumm N, Pestinger M, et al. Antibiotics in palliative medicine–Results from a prospective epidemiological investigation from the HOPE survey. Support Care Cancer 2012;20(2):325–333; doi: 10.1007/s00520-011-1084-1 21274577

[17] Rosenberg JH, Albrecht JS, Fromme EK, et al. Antimicrobial use for symptom management in patients receiving hospice and palliative care: A systematic review. J Palliat Med 2013;16(12):1568–1574; doi: 10.1089/jpm.2013.0276 24151960 PMC3868271

[18] Mohammed AA, Al-Zahrani AS, Sherisher MA, et al. The pattern of infection and antibiotics use in terminal cancer patients. J Egypt Natl Canc Inst 2014;26(3):147–152; doi: 10.1016/j.jnci.2014.05.002 25150130

[19] Baghban A, Juthani-Mehta M. Antimicrobial use at the end of life. Infect Dis Clin North Am 2017;31(4):639–647; doi: 10.1016/j.idc.2017.07.009 29079153

[20] Woo KY, Krasner DL, Kennedy B, et al. Palliative wound care management strategies for palliative patients and their circles of care. Adv Skin Wound Care 2015;28(3):130–142; doi: 10.1097/01.ASW.0000461116.13218.43 25679465

[21] Tilley CP, Fu MR, Van Cleeve J, et al. Symptoms of malignant fungating wounds and functional performance among patients with advanced cancer: An integrative review from 2000 to 2019. J Palliat Med 2020;23(6):848–862; doi: 10.1089/jpm.2019.0617 32349622

[22] Chrisman CA. Care of chronic wounds in palliative care and end-of-life patients. Int Wound J 2010;7(4):214–235; doi: 10.1111/j.1742-481X.2010.00682.x 20528993 PMC7951627

[23] Alvarez OM, Kalinski C, Nusbaum J, et al. Incorporating wound healing strategies to improve palliation (symptom management) in patients with chronic wounds. J Palliat Med 2007;10(5):1161–1189; doi: 10.1089/jpm.2007.9909 17985974

[24] Firmino F, Trotte LAC, Silva RS. Competências do Enfermeiro [Livro Eletrônico]: Especialista em Cuidados Paliativos no Brasil. 1ed. ANCP: São Paulo; 2022.

[25] Silva RS, Silva MJP. [Cap.1] A Enfermagem e os Cuidados Paliativos. In: Enfermagem em Cuidados Paliativos—Cuidando Para uma boa morte (Silva RS, Amaral JB, Malagutti W. eds.) Martinari: São Paulo; 2019.

[26] Howard KB, Bulechek GM, Dochterman JM, Wagner CM. NIC-Classificação das intervenções de enfermagem. In: Tradução de: Nursing Interventions Classification (NIC), 7 ed. (Imon de Oliveira TS. eds.) Elsevier: Rio de Janeiro; 2020.

[27] Freitas R D, Oliveira LCd, Mendes GLQ, et al. Barreiras para o encaminhamento para o cuidado paliativo exclusivo: A percepção do oncologista. Saúde Debate 2022;46(133):331–345; doi: 10.1590/0103-1104202213306

[28] Alcalde J, Zimmermann C. Stigma about palliative care: Origins and solutions. Ecancermedicalscience 2022;16:1377; doi: 10.3332/ecancer.2022.1377 35702413 PMC9116991

[29] Kaasa S, Loge JH, Aapro M, et al. Integration of oncology and palliative care: A Lancet oncology commission. Lancet Oncol 2018;19(11):e588–e653; doi: 10.1016/S1470-2045(18)30415-7 30344075

[30] Rosa RG, Goldani LZ. Cohort study of the impact of time to antibiotic administration on mortality in patients with febrile neutropenia. Antimicrob Agents Chemother 2014;58(7):3799–3803; doi: 10.1128/AAC.02561-14 24752269 PMC4068526

[31] Thompson AJ, Silveira MJ, Vitale CA, Malani PN. Antimicrobial use at the end of life among hospitalized patients with advanced cancer. Am J Hosp Palliat Care 2012;29(8):599–603; doi: 10.1177/1049909111432625 22218916

[32] Dyer J, Vaux L, Broom A, Broom J. Antimicrobial use in patients at the end of life in an Australian hospital. Infect Dis Health 2019;24(2):92–97; doi: 10.1016/j.idh.2018.12.001 30655096

[33] Spellberg B, Rice LB. Duration of antibiotic therapy: Shorter is better. Ann Intern Med 2019;171(3):210–211; doi: 10.7326/M19-1509 31284302 PMC6736742

[34] Arias-Fernández L, Suérez-Mier B, Martínez-Ortega MD, Lana A. Incidence and risk factors of phlebitis associated to peripheral intravenous catheters. Enferm Clin 2017;27(2):79–86; doi: 10.1016/j.enfcli.2016.07.008 27640931

[35] Salgueiro-Oliveira AdS, Basto ML, Braga LM, et al. Nursing practices in peripheral venous catheter: Phlebitis and patient safety. Texto Contexto - Enferm 2019;28:e20180109; doi: 10.1590/1980-265X-TCE-2018-0109

[36] Macedo F, Nunes C, Ladeira K, et al. Antimicrobial therapy in palliative care: An overview. Support Care Cancer 2018;26(5):1361–1367; doi: 10.1007/s00520-018-4090-8 29435712

[37] Hung KC, Lee LW, Liew YX, et al. Antibiotic Stewardship Program (ASP) in palliative care: Antibiotics, to give or not to give. Eur J Clin Microbiol Infect Dis 2022;41(1):29–36; doi: 10.1007/s10096-021-04325-z 34414518

[38] Lee S, O’Donovan L, Cohen AJ, et al. Prevalence and treatment of postobstructive pneumonia among older adults with advanced cancer. Antimicrob Steward Healthc Epidemiol 2022;2(1):e152; doi: 10.1017/ash.2022.293 36483405 PMC9726473

[39] Vitetta L, Kenner D, Sali A. Bacterial infections in terminally ill hospice patients. J Pain Symptom Manage 2000;20(5):326–334; doi: 10.1016/s0885-3924(00)00189-5 11068154

[40] Datta R, Zhu M, Han L, et al. Increased length of stay associated with antibiotic use in older adults with advanced cancer transitioned to comfort measures. Am J Hosp Palliat Care 2020;37(1):27–33; doi: 10.1177/1049909119855617 31185722 PMC6868290

[41] Moen MK, Løhre ET, Jakobsen G, et al. Antibiotic therapy in integrated oncology and palliative cancer care: An observational study. Cancers (Basel) 2022;14(7):1602; doi: 10.3390/cancers14071602 35406374 PMC8996984

[42] Prabhu RA, Salins N, Bharathi, Abraham, G. End of life care in end-stage kidney disease. Indian J Palliat Care 2021;27(Suppl 1):S37–S42; doi: 10.4103/ijpc.ijpc_64_21 34188377 PMC8191743

[43] Natuhwera G, Rabwoni M, Ellis P, Merriman A. Clinicians’ and nurses’ documentation practices in palliative and hospice care. Int J Palliat Nurs 2021;27(5):227–234; doi: 10.12968/ijpn.2021.27.5.227 34292773

[44] Hosoi T, Ozone S, Hamano J. Variations in vital signs at the end of life in non-cancer patients: A Retrospective Study. Ann Palliat Med 2020;9(5):2678–2683; doi: 10.21037/apm-20-1054 32819124

[45] Matsunami K, Tomita K, Touge H, et al. Physical signs and clinical findings before death in ill elderly patients. Am J Hosp Palliat Care 2018;35(4):712–717; doi: 10.1177/1049909117733661 28978223

[46] Sloane PD, Hoeffer B, Mitchell CM, et al. Effect of person-centered showering and the towel bath on bathing-associated aggression, agitation, and discomfort in nursing home residents with dementia: A randomized, controlled trial. J Am Geriatr Soc 2004;52(11):1795–1804; doi: 10.1111/j.1532-5415.2004.52501.x 15507054

[47] Hayashi E, Aoyama M, Masukawa K, et al. Bathing in terminal care of cancer patients and its relation to perceptions of a “good death”: A nationwide bereavement survey in Japan. Palliat Med Rep 2022;3(1):55–64; doi: 10.1089/pmr.2021.0075 35558866 PMC9081016

[48] Franco HCP, Stigar R, Souza SJP, Burci LM. Papel da enfermagem na equipe de cuidados paliativos: A humanização no processo de morte e morrer. Revista Gestão & Saúde 2017;17(2):48–61.

[49] Saint S, Trautner BW, Fowler KE, et al. A multicenter study of patient-reported infectious and noninfectious complications associated with indwelling urethral catheters. JAMA Intern Med 2018;178(8):1078–1085; doi: 10.1001/jamainternmed.2018.2417 29971436 PMC6143107

[50] Moataz A, Chadli A, Wichou E, et al. Facteurs prédictifs de l’inconfort lié à la sonde vésicale [Predictors of catheter-related bladder discomfort]. Prog Urol 2020;30(16):1045–1050; doi: 10.1016/j.purol.2020.09.014 33011083

[51] Pongprasobchai S, Jiranantakan T, Nimmannit A, Nopmaneejumruslers C. Comparison of the efficacy between lidocaine spray plus lidocaine jelly lubrication and lidocaine jelly lubrication alone prior to nasogastric intubation: A prospective double-blind randomized controlled study. J Med Assoc Thai 2007;90 Suppl 2:41–47. 19238647

[52] de Oliveira AS, Ribeiro CJN, Oliveira ALC, et al. Analgesic efficacy of 10% lidocaine spray during nasoenteral catheterization: Randomized triple-blind trial. Eur J Pain 2020;24(3):536–543; doi: 10.1002/ejp.1503 31705581

[53] Chang WP, Chen HM, Wu JR, et al. Adverse effects of non-intubated airway suctioning: A clinical data-based study. J Clin Nurs 2023;32(5-6):726–735; doi: 10.1111/jocn.16307 35347773

[54] Schneiderman L, De Ridder M. Chapter 14 - Medical futility. In: Handbook of Clinical Neurology, Vol. 118. (Bernat JL, Beresford HR. eds.) Elsevier; 2013; pp. 167–179. 10.1016/B978-0-444-53501-6.00014-7 24182376

[55] Oliveira AC, Silva MJP. Autonomia em cuidados paliativos: Conceitos e percepções de uma equipe de saúde. Acta Paul Enferm 2010;23(2):212–217.

[56] Dadalto L, Mascarenas IL, Matos AKH. Salvem também os idosos: Etarismo e a alocação de recursos na realidade brasileira de combate ao covid. Civilística.com 2020;9(2):1–19; Disponível: https://bit.ly/3nxL7oI [acesso 14 set 2020].

